# Antenatal Anovaginal Distance, a Potential Indicator of Perineal Damage during Pregnancy

**DOI:** 10.3390/healthcare12202044

**Published:** 2024-10-15

**Authors:** Federico Villani, Erich Cosmi, Zoe Lunardon, Martina Granci, Cristina Panizza, Barbara Mazzucato, Antonella Cavalieri, Mirela Marioara Toma, Roxana Furau, Cristian Furau

**Affiliations:** 1 Multidisciplinary Doctoral School, “Vasile Goldis” Western University of Arad, 310414 Arad, Romania; vllfrc@gmail.com; 2 Department of Women’s and Children’s Health, University of Padua, 35128 Padua, Italy; erich.cosmi@unipd.it (E.C.); martina.granci@unipd.it (M.G.); cristina.panizza@unipd.it (C.P.); 3 School of Medicine, University of Padua, 35128 Padua, Italy; zoe.lunardon@gmail.com; 4 The Rehabilitation Therapy of the Pelvic Floor, UniCamillus, Saint Camillus International University of Health and Medical Sciences, 00131 Rome, Italy; dr.ssa.barbara.mazzucato@gmail.com (B.M.); antonella.amaca@gmail.com (A.C.); 5 Department of Pharmacy, Faculty of Medicine and Pharmacy, University of Oradea, 410028 Oradea, Romania; 6 Department Medicine, Faculty of Medicine, “Vasile Goldis” Western University of Arad, 310414 Arad, Romania; roxanafurau@gmail.com; 7 Department of Pathophysiology, Faculty of Medicine, “Vasile Goldis” Western University of Arad, 310414 Arad, Romania

**Keywords:** perineal health, pregnancy, anovaginal distance, perineal tear, episiotomy, vaginal delivery

## Abstract

Background/Objectives: Perineal injuries, including episiotomies and spontaneous tears, are common complications during childbirth, often leading to significant discomfort and prolonged recovery for women. This retrospective observational cohort study aimed to explore the relationship between antenatal anovaginal distance (AVD) and the incidence of perineal injuries in a cohort of pregnant women evaluated for pelvic floor health at 28 to 32 weeks of gestation. Methods: Conducted at the University Hospital of Padua over 18 months, the study included 416 women who underwent vaginal delivery at term. Based on AVD, the study participants were divided into two groups: AVD-N group, which included 252 patients with AVD ≥ 2 cm, and the AVD-R group, which included 164 with AVD < 2 cm. The results of the pelvic floor assessment and those related to childbirth were then examined in relation to AVD (reduced vs. normal). Results: The study found that women with reduced AVD were more likely to experience perineal injuries. Specifically, the incidence of episiotomy and severe perineal tears (3rd and 4th degree) was significantly higher in the reduced AVD group (*p* < 0.05). Furthermore, a lower AVD was associated with increased perineal muscle hypertonicity and a higher likelihood of operative delivery with episiotomy. Logistic regression analysis confirmed that reduced AVD was an independent risk factor for perineal injuries, regardless of other maternal or neonatal characteristics. Conclusions: These results suggest that AVD measurement during pregnancy may help identify women at higher risk of perineal trauma, enabling more personalized obstetric care to mitigate these outcomes.

## 1. Introduction

Childbirth-related perineal trauma is a common complication during vaginal delivery, often leading to short-term and long-term maternal morbidity [1]. Perineal trauma refers to damage to the skin, muscles of the perineum, and the anal sphincter complex, including the anal epithelium, and typically results from either spontaneous laceration or an incision made during vaginal delivery [2]. These injuries are common outcomes after vaginal birth, affecting nearly 90% of women. Second-degree perineal tears are particularly prevalent, occurring twice as often in first-time births, with an incidence rate of around 40%. Obstetric anal sphincter injuries occur in approximately 3% of vaginal deliveries, with a significantly higher occurrence in first-time mothers compared to those who have previously given birth (6% vs. 2%) [3]. 

Several risk factors contribute to the occurrence of perineal trauma during childbirth. These include first-time vaginal births, maternal ethnicity (particularly Southeast Asian background), maternal age over 35 years, large birth weight or head circumference, fetal malposition, prolonged second stage of labor, and instrumental deliveries [4,5]. 

Perineal trauma can lead to a range of health issues with varying impacts on a woman’s quality of life. These issues include perineal pain, wound dehiscence, infections, dyspareunia, sexual dysfunction, and urinary or fecal incontinence [6,7,8,9]. Consequently, it is crucial for clinicians and midwives to provide evidence-based care to mitigate these physical and psychological symptoms.

The perineum, a soft tissue area extending from the anus to the posterior border of the vulvar vestibule, plays a critical role in childbirth. During pregnancy, increased blood flow occurs in this region, and the overstretching that can happen during vaginal delivery may result in trauma [10]. Perineal size and other anatomical parameters, including anovaginal distance (AVD) have recently emerged as potential anatomical predictors of perineal injury during childbirth. AVD is influenced by several factors, including maternal age, body mass index (BMI), and hormonal changes during pregnancy [11,12]. The perineum’s ability to accommodate the descending fetal head is essential in preventing tears, and a shorter perineum might predispose women to higher tension and thus, a higher injury risk [13]. Several studies have suggested that women with a shorter perineal length are more likely to undergo episiotomy and have a significantly higher risk of third- and fourth-degree perineal tears during vaginal delivery [14,15]. 

Despite these associations, there is limited data on how AVD measurements in the prenatal period correlate with perineal outcomes post-delivery. Previous studies have highlighted the importance of perineal body length and other anatomical parameters in predicting the likelihood of perineal tears during vaginal delivery [3,15,16,17]. However, most of these studies have focused on evaluating these parameters immediately before, during, or after childbirth—when intervention is no longer possible, and the opportunity to influence these outcomes has passed. Investigating the relationship between anovaginal distance (AVD) assessed during the gestational age of 28 to 32 weeks offers a valuable and underexplored window of opportunity. This period is early enough to identify women at higher risk of perineal trauma, allowing for the implementation of targeted prevention strategies before delivery. Such interventions could focus on improving muscle elasticity and promoting perineal stretching, thereby reducing the risk of tearing or the need for episiotomy during childbirth [7,18]. Moreover, understanding this relationship could enhance individualized risk assessment, enabling clinicians to tailor their management approaches more effectively. This study, therefore, seeks to fill a gap in the literature by evaluating the association between AVD and perineal outcomes, with the aim of contributing to improved strategies for preventing perineal trauma during childbirth.

## 2. Materials and Methods

### 2.1. Design and Patients

This observational retrospective cohort study was conducted at the Breastfeeding and Pelvic Floor Clinic of the University Hospital of Padua, a tertiary university hospital in Padua, Italy. This clinic specializes in the assessment and management of pelvic floor health and supports breastfeeding among new mothers. The study spanned 18 months, from 1 January 2023 to 31 June 2024, and included consecutive women who underwent perineal assessment at the University Hospital of Padua between 28 and 32 weeks of gestation and subsequently gave birth in the same facility between 37 + 0 and 41 + 6 weeks of gestation. Included in the analysis were all women with vaginal delivery at term. Both spontaneous vaginal deliveries and vacuum-assisted deliveries were included. Exclusion criteria were maternal age under 18 years, obesity (BMI > 30 kg/m^2^), diabetes, hypertension, preterm or post-term delivery, twin or multiple pregnancies, parity greater than 2, emergency or scheduled cesarean deliveries, macrosomia, and newborn head circumference >35 cm.

### 2.2. Data Collection

The study utilized data from a preexisting, prospectively maintained electronic database containing records of all deliveries at the hospital during the study period. The database included comprehensive information on patient history, labor and delivery parameters, and maternal and neonatal outcomes (Table 1). 

### 2.3. Study Protocol

The data of 472 patients who consecutively visited the clinic for pelvic floor evaluation and gave birth in the same clinic were checked for inclusion in the study. After applying the inclusion/exclusion criteria, 416 women were included in the final analysis. Based on the results obtained from AVD measurements in our study (from 0.5 cm to 4.0 cm) and data from the literature [15], we defined reduced AVD for values <2 cm and normal AVD for values ≥2 cm. According to AVD, the research subjects were split up into two groups: the AVD-N group, which included 252 patients with AVD ≥2 cm, and the AVD-R group, which included 164 with AVD <2 cm. The results of the pelvic floor assessment and those related to childbirth were then examined in relation to AVD (reduced vs. normal).

### 2.4. Statistical Analysis

Nominal variables are presented as frequencies and percentages, whereas continuous variables are reported as mean values with their corresponding standard deviations.

Data for nominal variables were presented as frequencies and percentages, whereas continuous variables were reported as mean values with their corresponding standard deviations. Associations between categorical variables related to maternal, pelvic, and obstetric characteristics were analyzed using the χ^2^ test. Quantitative variables were compared using either the two-sample *t*-test or the Mann-Whitney U test, depending on the Shapiro-Wilk test for normality. ANOVA (the Kruskal-Wallis non-parametric test) was used for analyzing the association between characteristics of the study population and AVD. Post hoc analyses were conducted using the Bonferroni test. A multivariate logistic regression model was developed to account for potential confounders in the association between AVD and perineal injuries. A *p*-value of ≤0.05 was regarded as the threshold for statistical significance. All analyses were conducted using JASP software (version 19).

## 3. Results

A total of 416 women, mean age 34.24 ± 3.93 (range 19–46), were included in this retrospective observational cohort study. The average gestational week at AVD assessment was 30.60 ± 1.58 (range 28–32) weeks, and the average AVD was 2.287 cm ± 0.809 (range 0.5–4 cm). The incidence of episiotomy in the entire group was 19.71%, and 242 (58.17%) suffered perineal tears of different degrees. Of the 416 women, 252 (60.58%) had normal AVD (≥2 cm) and 164 (39.42%) had reduced AVD (<2 cm). 

Results of the comparative analysis of the two groups, AVD-N (N = 252) and AVD-R (N = 164), across maternal and neonatal evaluated characteristics indicated that both groups had similar means of age and BMI, similar distributions of parity, and were evaluated around the same gestational week (*p* < 0.05). A significantly higher percentage of women in the AVD-N group had normal perineal tonicity (52.362%) compared to the AVD-R group (31.902%), while hypertonicity was significantly more common in the AVD-R group (63.42%) compared to the AVD-N group (38.89%) (*p* < 0.001).

Both groups had similar gestational ages at delivery, with no significant difference (*p* = 0.407). A higher percentage of women in the AVD-N group (57.29%) had a spontaneous vaginal delivery compared to the AVD-R group (43.29%), while OD with episiotomy was more common in the AVD-R group (20.12%) compared to the AVD-N group (6.34%) (*p* < 0.001).

The mean newborn weight and head circumference were very similar between the two groups, with no significant difference (*p* < 0.05). 

The AVD-R group had a higher rate of episiotomy (35.97%) compared to the AVD-N group (8.66%) (*p* < 0.001). A significant difference was also observed in the degree of tears at delivery. The AVD-N group had a higher percentage of no tears (49.20%) compared to the AVD-R group (30.49%), whereas 3rd and 4th-degree tears occurred only in the AVD-R group (*p* < 0.001) (Table 2).

**Figure 1:**
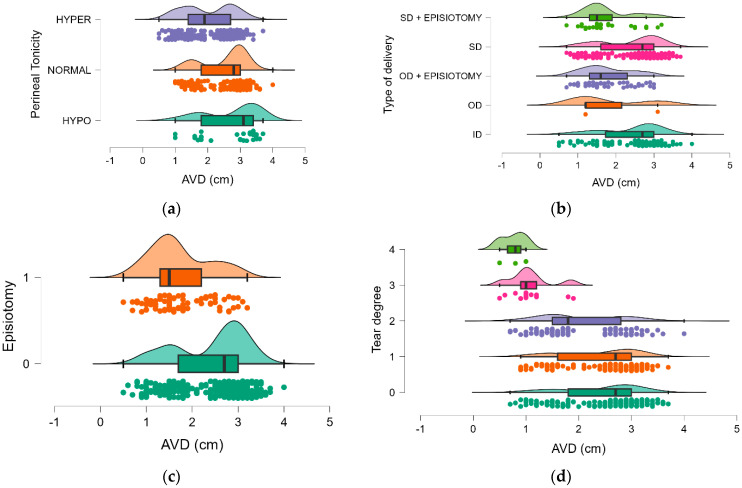
The distribution of AVD: (**a**)—by perineal tonicity, (**b**)—by type of delivery, (**c**)—by tear degree, (**d**)—by episiotomy.

Descriptive analysis of the AVD based on different perineal characteristics, types of delivery, and perineal injuries showed that AVD is generally higher in spontaneous (SD) and induced (ID) deliveries compared to operative deliveries (OD); the presence of an episiotomy in spontaneous deliveries appears to lower the AVD (mean = 1.748) compared to when no episiotomy is performed (mean = 2.404); and AVD decreases with increasing severity of perineal laceration, from grade 0 to grade 4. The most severe lacerations (grade 3 and 4) are associated with the lowest AVD values, indicating a significant impact of AVD on severe perineal injuries. All results are presented in Table 3 and Figure 1.

Data were further examined using the Kruskal-Wallis test, with Bonferroni post hoc comparison, to determine whether AVD and parameters with a significant difference between the two groups were associated. To achieve this, AVD was used as a continuous variable. There were statistically significant AVD-related differences for all tested parameters (*p* < 0.05). At the level of perineal tonicity, significant differences in AVD were observed between the hypo and hyper groups, (0.738 cm, *p* < 0.001) and between the normal and hyper groups (0.531 cm, *p* < 0.001), suggesting that individuals with hypertonic perineal muscles have a significantly smaller AVD compared to those with hypotonic or normal tonicity. There is no significant difference between the hypo and normal groups (0.208 cm, *p* = 0.358), indicating that the AVDs are more similar between these two groups.

Regarding the type of delivery, the significant differences were primarily between deliveries with and without episiotomy. ID has a significantly higher AVD compared to OD + episiotomy and SD + episiotomy, suggesting that episiotomy is associated with a reduction in AVD. SD also shows a higher AVD compared to SD with episiotomy, further reinforcing that a lower AVD leads to episiotomy. There are no significant differences between ID and OD, ID and SD, OD and OD + episiotomy, OD and SD, and OD + episiotomy and SD + episiotomy. 

The mean AVD was higher in the group without episiotomy (2.427 cm) compared to the group with episiotomy (1.718 cm). This indicates that the anovaginal distance is generally shorter when an episiotomy is performed. The results indicate that there are significant differences in AVD as the degree of perineal tear increases from 0 to 4 (*p* < 0.05). Particularly, larger differences in AVD were registered as the tear degree increases, with tear degrees 3 and 4 associated with much lower AVD compared to lower degrees. The non-significant results between tear degrees 0 and 1 Mean Difference = 0.035, *p*-value = 1.000), as well as between 3 and 4 Mean Difference = 0.310, *p*-value = 1.000) suggest that AVD does not change as much between these adjacent degrees (Table 4).

Two logistic regression analyses were conducted, incorporating all relevant factors as independent variables and perineal injury (episiotomy and perineal tears) as dependent variables. Continuous variables included age, BMI, newborn weight (g), and newborn head circumference (cm). The categorical variables were assigned specific numerical values for analysis: episiotomy: 0 = no episiotomy, 1 = episiotomy, tear degree 0 = no (laceration grades 0 and 1), 1 = yes (laceration grades 2, 3 and 4), AVD: <2 = reduced, AVD ≥2 cm = normal. 

After adjusting for confounding factors, the odds ratio (OR) remained statistically significant at 3.588 (95% CI 0.972–1.120) for predicting perineal tears and 6.346 (95% CI 3.560–11.311) for episiotomy (Table 5). The analysis highlights that reduced AVD is a significant risk factor for both perineal tears and episiotomy. It has a strong effect on increasing the likelihood of these injuries, independent of other factors such as age, BMI, and newborn characteristics.

## 4. Discussion

Despite extensive research, the effectiveness of interventions to reduce the risk of perineal trauma remains controversial. Understanding the factors that contribute to these injuries is crucial for improving maternal outcomes and guiding clinical practice. Factors such as fetal weight, maternal age, episiotomy, and labor duration have traditionally been studied as predictors of perineal injury [16]. Recently, attention has turned to anatomical measurements, including the AVD [15].

This study aimed to investigate the anovaginal distance (AVD) measured between 28–32 weeks of gestation as a potential determinant of perineal injuries during childbirth. 

Our results indicate significant differences between women with normal AVD (AVD-N) and those with reduced AVD (AVD-R) across several key outcomes, particularly regarding perineal tonicity, mode of delivery, and the severity of perineal injuries, suggesting that measuring the anogenital distance at 28–32 weeks of gestation may offer a non-invasive method to predict perineal injury risk during childbirth, potentially guiding interventions to improve maternal outcomes. The gestational age between 28 and 32 weeks was recommended for measuring the AVD because in this period the tissues are still responsive to physiological changes but are not yet under the full stress related to late pregnancy. This gestational age allows a time window that is early enough for prevention strategies to be implemented if high-risk findings are detected.

The study involving 416 women provides insightful data on the relationship between AVD and perineal outcomes during childbirth. The average AVD in the cohort was 2.287 cm, and 60.58% of the women had a normal AVD (≥2 cm), while 39.42% had a reduced AVD (<2 cm). 

Comparative analysis showed that women with a reduced AVD were more likely to experience perineal hypertonicity, operative deliveries with episiotomy, and severe perineal tears compared to those with a normal AVD. 

In this study, individuals with hypertonic perineal muscles have significantly smaller AVD compared to those with hypotonic or normal tonicity. Pelvic floor hypertonicity occurs when the muscles of the pelvic floor are excessively tense and cannot fully relax. This condition can lead to various pelvic health issues, including constipation, painful intercourse (dyspareunia), urinary urgency, and chronic pelvic pain, and can have a significant impact on a woman’s overall pelvic health and quality of life [20]. According to previous studies, women with higher degrees of dyspareunia are more likely to have third-degree tears during childbirth. This has been linked to the inability of some patients to relax their pelvic floor muscles, suggesting that proper pelvic floor relaxation plays an important role in childbirth [20,21]. These findings highlight the importance of pelvic floor assessment and targeted interventions, such as physical therapy, to help women with hypertonic pelvic floors achieve better muscle relaxation, ultimately supporting safer and less traumatic childbirth experiences. 

The AVD-R group has been reregistered a lower rate of spontaneous vaginal deliveries and a higher rate of operative deliveries with episiotomy, showing that a shorter AVD is associated with higher rates of episiotomy and instrumental delivery. Also, subjects with reduced AVD had a significantly higher rate of episiotomy (35.97%) and severe lacerations, including third- and fourth-degree tears, than the AVD-N group. Previous studies have highlighted the importance of perineal body length and other anatomical parameters in predicting the likelihood of perineal tears during vaginal delivery and identified the length of the perineal body as a significant predictor of obstetric anal sphincter injuries (OASIS) [22,23,24,25].

The logistic regression analysis further reinforces this relationship, showing that a reduced AVD is a potential predictor of perineal injuries, including both perineal tears and episiotomy, independent of other factors like age, BMI, and newborn characteristics. 

The long-term consequences of these injuries can include pelvic floor dysfunction, leading to urinary or fecal incontinence and pelvic organ prolapse. Women may also experience chronic perineal pain and dyspareunia, negatively affecting their sexual health and overall quality of life [26]. These physical symptoms can contribute to emotional distress, anxiety, and depression, further complicating postpartum recovery and reducing long-term well-being [27,28].

Clinically, assessing AVD during pregnancy, particularly between 28 and 32 weeks, allows for early identification of women at higher risk of perineal trauma. This enables the implementation of targeted prevention strategies, such as pelvic floor training to improve muscle elasticity, antenatal perineal massage to enhance flexibility, and, in some cases, considering alternative delivery methods to minimize trauma. Evidence suggests that starting perineal massage in the third trimester can improve muscle elasticity and aid in perineal stretching during childbirth, potentially reducing the likelihood of tearing or the need for an episiotomy [3,7]. Also, perineal support techniques or alternative birthing positions could be considered to mitigate the risk of injury [3]. Additionally, the use of episiotomy for women with reduced AVD should be based on individualized clinical judgment rather than routine practice. Early episiotomy based solely on reduced AVD presents ethical considerations, as it is an invasive procedure with associated risks such as increased pain, infection, and extended recovery time [29,30]. For women who do sustain perineal injuries, focused postpartum rehabilitation can aid in restoring function and alleviating long-term complications [26]. 

By incorporating AVD measurement into prenatal care, clinicians can improve maternal outcomes and enhance personalized obstetric management. However, our study serves as a preliminary step in understanding the relationship between AVD and perineal injury, and future research should aim to refine this understanding to establish standardized protocols that integrate AVD into clinical decision-making.

This study offers an innovative approach by exploring anovaginal distance (AVD) as a non-invasive predictor of perineal trauma during childbirth. Measuring AVD between 28 and 32 weeks of gestation provides an early opportunity to identify women at high risk of perineal injuries, allowing for timely implementation of preventive strategies. The use of robust statistical analysis strengthens the findings, demonstrating that reduced AVD is associated with a higher risk of episiotomy and severe perineal tears, independent of other factors. Clinically, the study highlights the potential to incorporate AVD measurement into prenatal care, supporting more personalized obstetric management.

Despite the promising findings, the retrospective nature of the study limits its capacity to establish definitive causal relationships and introduces potential biases, such as selection bias and recall bias, and important confounding factors like childbirth position, maternal nutrition, and pelvic floor exercises were not controlled for, which could have influenced the outcomes. Although we employed a standardized protocol for AVD, potential variability in these measurements exists, and further steps to ensure measurement reliability should be taken in future studies. Additionally, the exclusion of women with a BMI greater than 30 restricts the applicability of the results, as obesity is a known risk factor for perineal trauma. Also, the study’s geographic limitation to a single hospital may restrict the generalizability of the findings, requiring broader validation in diverse settings. The lack of standardized protocols for integrating AVD measurement into clinical practice highlights the need for further research to establish its role in routine obstetric care. Lastly, while the study touches on long-term consequences of perineal trauma, future longitudinal studies are necessary to assess these impacts more comprehensively.

This study aims to raise awareness about the prevention of perineal trauma, an issue that remains largely overlooked in obstetrics. Currently, there are no specific guidelines addressing this issue, but we hope that our findings will inspire larger studies that incorporate a more diverse population, control for additional confounders, ensure measurement consistency, and explore long-term outcomes and tailored clinical strategies.

## 5. Conclusions

This study suggested that early assessment of AVD could serve as a valuable clinical tool for identifying women at risk of severe perineal trauma, allowing for the implementation of personalized preventive strategies or targeted interventions to improve pelvic floor elasticity. The significant number of women with perineal tears and the varying AVD measurements emphasize the need for further research into how AVD can be used to optimize childbirth outcomes, possibly leading to more individualized care plans that reduce the risk of perineal injury.

## Figures and Tables

**Table 1:** Maternal characteristics, delivery, and neonatal outcomes considered for the study.

Maternal Characteristics
Age: Recorded in years at the initial prenatal visit
BMI: Calculated from height (cm) and weight (kg) at the initial prenatal visit. BMI was categorized as follows: ⚬ Underweight: <18.5 (combined with normal weight due to small numbers) ⚬ Normal weight: 18.5 to 24.9 ⚬ Overweight: 25 to 29.9 ⚬ Obese: ≥30 In this study obese patients have been excluded, but lightly underweight patients (BMI 16.5–18.5 kg/m^2^) have been included. Due to a small sample size, the underweight and normal weight categories were combined.
Parity: Recorded as the number of previous completed pregnancies and categorized as either nulliparous (a woman experiencing her first pregnancy and delivery) or secundiparous (a woman expecting her second child).
Anovaginal distance (AVD): Recorded as the distance between posterior vaginal fourchette and the upper edge of the anal orifice. All assessments were performed by trained obstetricians experienced in perineal evaluation, following the same protocol. Each measurement was performed using a standardized disposable paper ruler, and all patients were positioned in the dorsal lithotomy position to minimize variation. The ruler was placed with the starting edge (zero) placed exactly on the lower margin of the vaginal fourchette and oriented vertically along the sagittal line of the perineum towards the center of the anal orifice. The nearest tenth of a centimeter corresponding on the ruler with the upper edge of the anal orifice was recorded as the AVD. After the primary measurement, a second trained observer independently verified the result. In cases where the discrepancy between the two measurements exceeded 0.2 cm, the measurement was repeated until agreement was reached.
Perineal Tonicity is one of the parameters that is assessed during a pelvic floor evaluation. We discriminate between a hypertonic perineum, a hypotonic perineum, and normal perineal tonicity. This parameter was assessed by an experienced obstetrician using both external and internal palpatory techniques, following standardized guidelines from the Italian Society of Gynecology and Obstetrics (SIGO) and the Italian Association of Obstetricians and Gynecologists (AOGOI). External tone was evaluated by applying light pressure on the perineal tendinous center to assess resistance, while internal tone was assessed via a vaginal examination by applying downward pressure. All assessments were performed by trained obstetricians experienced in perineal evaluation, following the same protocol outlined in the AOGOI guidelines [19].
Gestational Week (GW) = GW at pelvic floor evaluation (GW at AVD measured between 28 and 32 weeks) and GW at delivery (only patients who gave birth between 37 + 0 and 42 + 0 GW have been included in the study).
Delivery outcome
Type of delivery ⚬ SD: spontaneous labor and delivery (natural birth) ⚬ ID: induced labor and delivery ⚬ OD: Operative or instrumental delivery (fetal extraction using vacuum extractors)
Episiotomy: An incision made in the posterior vaginal wall and part of the perineum to enlarge the vaginal opening, facilitating delivery and reducing the risk of lacerations. In the database, episiotomy is associated with both spontaneous (SVD + episiotomy) and operative deliveries (OVD + episiotomy).
Perineal tears or lacerations are defined as the perineal trauma suffered by the patient as a consequence of the passing of the fetus through the birth canal. Spontaneous tears are divided into 4 categories: 1st, 2nd, 3rd, and 4th degree tears, according to the depth of the tear in the perineum, as diagnosed by an obstetrician or midwife at the time of birth.
Neonatal outcomes
Newborn Weight—recorded in grams, measured after birth
Newborn head circumference—recorded in centimeters, measured after birth

**Table 2:** Associations of demographic and clinical characteristics with reduced AVD.

Characteristic	AVD-N (N = 252)	AVD-R (N = 164)	*p*
Maternal age (years)	34.01 (SD = 4.009)	34.585 (SD = 3.795)	0.096
Maternal BMI (kg/m^2^)	21.756 (SD = 2.915)	21.951 (SD = 2.753)	0.410
Underweight/normal weight	221 (87.70%)	143 (87.19%)	0.638
Overweight	31 (12.30%)	21 (12.81%)	
Parity			
0	199 (78.97%)	131 (79.88%)	0.823
1	53 (21.03%)	33 (20.12%)	
GW at AVD measured	30.556 (SD = 1.577)	30.659 (SD = 1.584)	0.407
28	47 (18.65%)	29 (17.68%)	0.693
28	27 (10.71%)	16 (9.75%)	
30	30 (11.91%)	20 (12.20%)	
31	35 (13.90%)	16 (9.75%)	
32	113 (44.84%)	83 (50.61%)	
AVD	2.881 (SD = 0.340)	1.375 (SD = 0.330)	<0.001
Perineal tonicity			
Hypo	20 (7.94%)	9 (5.488%)	<0.001
Normal	134 (53.17%)	51 (31.10%)	
Hyper	98 (38.89%)	104 (63.42%)	
Mode of delivery			
Spontan delivery	146 (57.29%)	71 (43.29%)	<0.001
Spontan delivery +episiotomy	7 (2.78%)	22 (13.42%)	
Operative delivery	1 (0.40%)	2 (1.22%)	
Operative delivery + episiotomy	16 (6.34%)	33 (20.12%)	
Induced delivery	82 (32.54%)	36 (21.95%)	
GW at delivery	39.42 (SD = 1.100)	39.29 (SD = 1.12)	0.222
<40 weeks’ gestation (37–39)	122 (48.41%)	80 (48.78%)	0.251
≥40 weeks’ gestation (40–41)	130 (51.59%)	84 (51.22%)	
Newborn weight (g)	3304.159 (SD = 365.760)	3290.634 (SD = 402.988)	0.868
Newborn head circumference (cm)	34.269 (SD = 1.220)	34.367 (SD = 1.298)	0.373
Episiotomy			
yes	23.00 (8.66%)	59 (35.97%)	<0.001
no	229 (91.34%)	105 (64.03%)	
Tear degree at delivery			
Laceration grade 0	124 (49.20%)	50 (30.49%)	<0.001
Laceration grade 1	85 (33.73%)	38 (23.17%)	
Laceration grade 2	43 (17.06%)	60 (36.59%)	
Laceration grade 3	0 (0%)	13 (7.92%)	
Laceration grade 4	0 (0%)	3 (1.63%)	

**Table 3:** Descriptive analysis of AVD based on perineal tonicity, type of delivery and perineal injuries.

Variable	AVD					
	N	Median	Mean	Std. Deviation	Minimum	Maximum
Perineal tonicity						
Hypo	29	3.100	2.738	0.868	1.000	3.700
Normal	185	2.800	2.530	0.711	1.000	4.000
Hyper	202	1.900	2.000	0.786	0.500	3.700
Type of delivery						
SD	217	2.700	2.404	0.785	0.700	3.700
SD + EPISIOTOMY	29	1.500	1.748	0.649	0.700	3.200
OD	3	1.200	1.833	1.097	1.200	3.100
OD + EPISIOTOMY	49	1.600	1.759	0.642	0.700	3.000
ID	118	2.700	2.435	0.809	0.500	4.000
Episiotomy						
No	334	2.700	2.427	0.784	0.500	4.000
Yes	82	1.500	1.718	0.651	0.500	3.200
Tear dedree						
Laceration grade 0	174	2.700	2.448	0.748	0.700	3.700
Laceration grade 1	123	2.700	2.413	0.757	0.900	3.700
Laceration grade 2	103	1.800	2.061	0.801	0.700	4.000
Laceration grade 3	13	1.000	1.077	0.400	0.500	1.900
Laceration grade 4	3	0.800	0.767	0.252	0.500	1.000

**Table 4:** Post hoc comparisons for association between AVD, perineal tonicity, type of delivery, episiotomy, and tear degree.

Parameters		Mean Difference	t	pbonf
Perineal tonicity				
Hypo	Normal	0.208	1.369	0.515
	Hyper	0.738	4.895	<0.001
Normal	Hyper	0.531	6.866	<0.001
Type of delivery				
ID	OD	0.601	1.336	1.000
	OD + E	0.676	5.163	<0.001
	SD	0.031	0.347	1.000
	SD + E	0.686	4.302	<0.001
OD	OD + E	0.074	0.162	1.000
	SD	−0.571	−1.275	1.000
	SD + E	0.085	0.182	1.000
OD + E	SD	−0.645	−5.297	<0.001
	SD + E	0.011	0.060	1.000
SD	SD + E	0.656	4.309	<0.001
Episiotomy				
Episiotomy	Yes/not	0.708	7.568	<0.001
Tear degree				
Laceration grade 0	1	0.035	0.397	1.000
	2	0.387	4.125	<0.001
	3	1.371	6.319	<0.001
	4	1.682	3.826	0.002
Laceration grade 1	2	0.352	3.490	0.005
	3	1.336	6.070	<0.001
	4	1.646	3.733	0.002
Laceration grade 2	3	0.984	4.430	<0.001
	4	1.294	2.928	0.036
Laceration grade 3	4	0.310	0.642	1.000

**Table 5:** Associations of AVD category with perineal tears and episiotomy.

Factor	Dependent Variable	Coeficients	Wald	*p*	OR	95% CI (OR)	
						Lower	Upper
Age (years)	PT	−0.040	1.868	0.172	0.961	3.560	11.311
	E	0.042	1.348	0.246	1.043	0.972	1.120
AVD (REDUCED)	PT	1.277	24.597	<0.001	3.585	0.972	1.120
	E	1.848	39.259	<0.001	6.3461	3.560	11.311
BMI	PT	0.006	0.020	0.887	1.006	0.956	1.150
	E	0.047	0.998	0.318	1.048	0.956	1.150
Perineal Tonicity (NORMAL)	PT	0.012	7.676 × 10^−4^	0.978	1.012	0.555	13.095
	E	0.991	1.511	0.219	2.695	0.555	13.095
Perineal Tonicity (HYPER)	PT	0.310	0.497	0.481	1.363	0.357	8.351
	E	0.547	0.463	0.496	1.728	0.357	8.351
Newborn weight (g)	PT	0.000	1.756	0.185	1.000	0.999	1.001
	E	0.000	0.012	0.914	1.000	0.999	1.001
Newborn head circumference (cm)	PT	−0.015	0.019	0.891	0.985	1.024	1.737
	E	0.288	4.567	0.033	1.334	1.024	1.737
PARA (1001)	PT	−0.546	3.828	0.050	0.579	0.105	0.623
	E	−1.362	9.015	0.003	0.256	0.105	0.623
Type of delivery (OD)	PT	2.678	39.867	<0.001	0.069	0.000	0.001
	E	7.243	51.090	<0.001	1398.048	191.863	10,187.160

PT—perineal tears, E—episiotomy.

## Data Availability

The original contributions presented in the study are included in the article, further inquiries can be directed to the corresponding authors.
